# Glucose-lowering effect of Reducose® enriched with 1-deoxynojirimycin and l-leucine: Studies on insulin secretion in INS-1 cells and reduction of blood glucose in diabetic rats

**DOI:** 10.1016/j.heliyon.2024.e25499

**Published:** 2024-02-01

**Authors:** Dahae Lee, Ji Yun Baek, Ye Jung Choi, Min Ji Han, Seon Hwa Kim, Tae Hoon Kim, Sanghyun Lee, Ki Sung Kang

**Affiliations:** a College of Korean Medicine, Gachon University, Seongnam, 13120, Republic of Korea; b Vixxol Corporation, Gunpo, 15807, Republic of Korea; c Department of Plant Science and Technology, Chung-Ang University, Anseong, 17546, Republic of Korea

**Keywords:** Reducose®, 1-Deoxynojirimycin, l-leucine, Insulin secretion, Glucose

## Abstract

The extract of mulberry leaf and its active ingredients have already been reported to have anti-diabetic effects; however, further studies are required to obtain better quality extracts and higher yields of active ingredients. Reducose® is a commercially available aqueous extract of mulberry leaves with a high content of active ingredients. In this study, the biological activities of Reducose®, 1-deoxynojirimycin, and l-leucine were evaluated using a glucose-stimulated insulin secretion (GSIS) assay. The GSIS assay results were expressed as the glucose-stimulated index (GSI). Considering the pharmacological safety in pancreatic β-cells, the appropriate non-toxic concentrations were selected by screening for cytotoxicity of Reducose®, 1-deoxynojirimycin, and l-leucine before the GSIS assay. The effect of Reducose®, 1-deoxynojirimycin, and l-leucine on glucose-stimulated insulin secretion in INS-1 cells was compared. Reducose®, 1-deoxynojirimycin, and l-leucine increased the GSI values more effectively than gliclazide (positive control). This was associated with an increase in protein expression, such as peroxisome proliferator-activated receptor-γ, insulin receptor substrate-2, activated pancreatic and duodenal homeobox-1, which are related to the regulation of pancreatic β-cell function and survival. In order to elucidate the effect of Reducose® in anti-diabetic effects, blood glucose levels, insulin levels, and liver and lipid concentrations were investigated in a Sprague-Dawley rat model of high-fat diet/streptozotocin-induced diabetes. We observed that administration of Reducose® can decrease fasting blood glucose levels and reduce the production of AST, ALT, TG, and TC to a similar extent as metformin (positive control). These results suggested that Reducose® play a role in promoting GSIS but not enough to show that the content and proportion of 1-deoxynojirimycin and l-leucine play an important role in the GSIS activity of Reducose®.

## 1 Introduction

Approximately 90 % of all diabetes cases are type 2 diabetes mellitus (T2DM), which is a multifactorial disease characterized by persistent hyperglycemia caused by pancreatic β-cell dysfunction and abnormal insulin action and secretion [[1], [2], [3]]. The total number of people with diabetes is expected to increase from 171 million in 2000 to approximately 366 million by 2030 [4,5]. Uncontrolled T2DM is a serious medical condition because it can lead to serious chronic complications, such as slow-healing wounds and kidney or eye diseases [6,7]. The number of patients with T2DM continues to rise, and given that it is closely related to lifestyle, numerous therapeutic options have been developed; however, few patients still achieve long-term glycemic control [8]. There are three main treatment approaches for T2DM that improve hyperglycemia. An increase in glucose-stimulated insulin secretion (GSIS) using insulin secretagogues, an increase in the use of insulin sensitizers for insulin action, and a decrease in the need for insulin using glucose absorption inhibitors [9].

Glucose homeostasis is important in the development of T2DM and is maintained by insulin secretion from pancreatic cells in response to blood glucose levels [10]. Sulfonylureas, oral insulinotropic agents first introduced in the 1950s, increase insulin secretion from pancreatic cells by closing ATP-dependent potassium (K_ATP_) channels located on the cell membrane [11]. Drugs in this class cause hypoglycemia by continuously stimulating insulin secretion irrespective of the blood sugar level [12]. Dipeptidyl peptidase-4 (DPP-4) inhibitors have recently been developed to compensate for this shortcoming; DPP-4 inhibitors increase insulin secretion but are glucose-dependent [13]. Recently, DPP-4 inhibitors have been reported to be associated with an increased risk of joint pain [14]. Therefore, continuous efforts are required to develop improved therapies to correct hypoglycemia.

The anti-diabetic efficacy of mulberry (Moraceae, *Morus alba* L.) has long been known, and many studies have shown that 1-deoxynojirimycin, an α-glucosidase inhibitor, is responsible for this effect [15]. Reducose® is a commercially available aqueous extract of mulberry leaves. The 1-deoxynojirimycin content of common mulberry leaf extract is approximately 0.1 %, and the 1-deoxynojirimycin content in Reducose® is 5 %, which is approximately 50-times higher. Additionally, it is characterized by the presence of approximately 5 % l-leucine, which activates insulin secretion. Reducose® can be used as a dietary supplement or directly blended into functional foods and drinks. Several randomized, placebo-controlled studies have demonstrated the efficacy and safety of Reducose® [[16], [17], [18]].

In an effort to discover promising sources for novel anti-diabetic agents derived from natural products, the effect of Reducose®, 1-deoxynojirimycin, and l-leucine on insulin secretion was screened in INS-1 rat pancreatic β-cells using a GSIS assay. From the preliminary screening, Reducose®, 1-deoxynojirimycin, and l-leucine increased the glucose stimulated index (GSI) values more effectively than gliclazide (positive control). Furthermore, we investigated the change in protein expression related to the regulation of pancreatic β-cell function and survival after treatment with Reducose®, 1-deoxynojirimycin, and l-leucine. Additionally, we further examined the biological effects of Reducose® using a rat model of high-fat diet/streptozotocin-induced diabetes to explore its effects in vivo.

## 2 Materials and methods

### 2.1 Plant material

Reducose**®** is manufactured by Phynova Group Ltd. (Long Hanborough, UK) and supplied by Vixxol (Gunpo, South Korea). Briefly, Reducose**®** is prepared from mulberry (Moraceae, Morus alba L.) as reported previously [[16], [17], [18]], and contains 5 % 1-deoxynojirimycin and l-leucine. The 1-deoxynojirimycin and l-leucine were purchased from Sigma-Aldrich (Inc. St. Louis, MO. USA).

### 2.2 Cell culture

The INS-1 rat pancreatic β-cells were obtained from the Biohermes, Shanghai, China, and cultivated in Roswell Park Memorial Institute (RPMI) 1640 medium (Cellgro, Manassas, VA, USA) supplemented with 10 % fetal bovine serum (FBS), 1 % penicillin/streptomycin (Invitrogen Co., Grand Island, NY, USA), 2 mM l-glutamine, 0.05 mM 2-mercaptoethanol, 11 mM d-glucose, 10 mM HEPES, and 1 mM sodium pyruvate in a CO_2_ incubator containing 5 % CO_2_ at 37 °C.

### 2.3 Ez-Cytox cell viability assay

The effects of the extracts, fractions, and compounds on the viability of INS-1 cells were measured using an Ez-Cytox cell viability assay kit (Daeil Lab Service Co., Seoul, Korea) to determine the concentrations that were non-toxic to INS-1 cells. Briefly, INS-1 cells (1.5 × 10 ^4^ cells/well) were seeded into 96-well plates, incubated for 24 h, and subsequently treated with the extracts, fractions, or compounds for 24 h. That was followed by the addition of 10 % (v/v) Ez-Cytox reagent for 2 h, and the optical densities (ODs) were measured at 450 nm with a microplate reader (PowerWave XS, Bio-Tek Instruments, Winooski, VT, USA).

### 2.4 GSIS assay

The effects of the extracts, fractions, and compounds on GSIS in INS-1 cells were measured using a rat insulin ELISA kit (Gentaur, Shibayagi Co. Ltd., Gunma, Shibukaw, Japan). Briefly, INS-1 cells (2 × 10 ^5^ cells/well) were seeded in 12-well plates and incubated for 24 h. To reproduce basal conditions, INS-1 cells were washed twice with Krebs-Ringer bicarbonate buffer (KRB, pH 7.4) containing 3.3 mM glucose. After incubation in fresh KRB containing 3.3 mM glucose for 1 h to simulate starvation conditions, INS-1 cells were pre-treated in a KRB-containing extract, fraction, compound, or gliclazide for 30 min, followed by a 1 h simulation with 3.3 mM low-glucose KRB solution or 16.7 mM high-glucose KRB solution. GSIS was measured using the collected supernatants in accordance with the manufacturer's instructions. The OD was measured at 450 nm using a microplate reader (PowerWave XS; Bio-Tek Instruments, Winooski, VT, USA). GSIS was expressed as a GSI (insulin concentration at 16.7 mM glucose/insulin concentration at 3.3 mM glucose).

### 2.5 Western blotting

For Western blot analysis, INS-1 cells (4.75 × 10 ^5^ cells/well) were seeded in 6-well plates and incubated for 24 h, followed by treatment with the compounds for 24 h. Total protein was extracted by the addition of RIPA buffer (Cell Signaling, Danvers, MA, USA) containing 1 mM phenylmethylsulfonyl fluoride for 20 min at 4 °C. This was followed by 10 % sodium dodecyl sulfate-polyacrylamide gel electrophoresis. Proteins were transferred onto polyvinylidene difluoride membranes by electroplating. The membrane was probed with the primary antibodies against peroxisome proliferator-activated receptor-γ (PPARγ), insulin receptor substrate-2 (IRS-2) (Ser731), IRS-2, pancreatic and duodenal homeobox-1 (PDX-1), and glyceraldehyde 3-phosphate dehydrogenase (GAPDH), followed by horseradish peroxidase (HRP)-conjugated anti-rabbit secondary antibody. ECL Plus Western blotting detection reagents (GE Healthcare, Little Chalfont, UK) were used to visualize the probed membranes, which were analyzed using a chemiluminescence system (FUSION Solo, PEQLAB Biotechnologie GmbH, Erlangen, Germany).

### 2.6 Animal care and diabetes induction

**Fig. 1:**
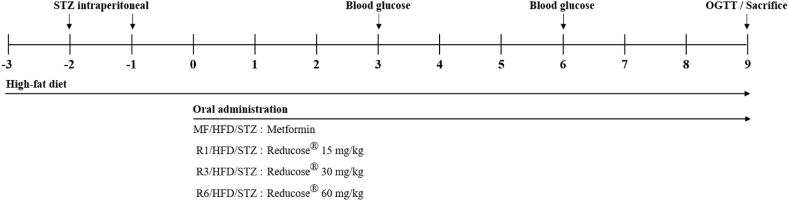
Model of STZ-induced diabetic rat.

Male Sprague Dawley rats at 5 weeks of age were purchased from BioLink and acclimated to the animal housing facility for one week with ad libitum access to food and water. All animals were fed a high-fat diet (D12492, 60 % kcal from fat, Research Diet Inc, USA) and one week later, diabetes was induced by intraperitoneal injection of streptozotocin (STZ; Sigma, USA, 30 mg/kg in 0.1 M citric acid buffer, pH 4.5) at a dose of 1 mL/kg, administered twice with a one-week interval. After diabetes induction, the animals were divided into groups based on blood glucose levels measured from tail vein samples, with each group consisting of 8 rats. Reducose® administration was performed once daily via oral gavage, with the appropriate dose dissolved in distilled water. Throughout the study, body weight and food intake were measured weekly, and at the end of the experiment, animals were sacrificed to collect epididymal fat for weight measurement. The experimental details are summarized in Fig. 1. The study was approved by institutional animal care and use committees of Gachon University (GU1-2022-IA0068).

### 2.7 Oral glucose tolerance test (OGTT)

The oral glucose tolerance test was conducted in the 9th week after the start of sample administration. Prior to the experiment, a 16-h fasting period was observed, and glucose (D-(+)-Glucose, Sigma, USA) was administered orally at a dose of 2 g/kg. Blood samples were collected from the tail vein at 0 min, 30 min, 60 min, and 120 min intervals, and blood glucose levels were measured using a blood glucose monitoring system (Accu check, Germany). GraphPad Prism 5.00 software was used to obtain the area under the curve (AUC) to obtain the AUC of OGTT.

### 2.8 Biochemical assay

Prior to sacrificing the animals at the end of the experimental period, they were fasted for 12 h, and blood samples were collected from the abdominal aorta. The collected blood samples were centrifuged at 2000 G, 4 °C for 15 min to obtain serum, which was then collected and stored at −70 °C until further analysis. Serum aspartate aminotransferase (AST), alanine aminotransferase (ALT), triglyceride (TG) and total cholesterol (TC), and glucose levels were analyzed by a specialized testing institution, GENIA (Seongnam, Korea). Insulin levels were analyzed using a rat insulin ELISA kit according to the instructions provided in the kit manual.

### 2.9 Statistical analysis

Statistical analysis was performed using GraphPad PRISM statistical package (version 5.00, GraphPad Software Inc., San Diego, USA). To assess the significance between groups, one-way analysis of variance (ANOVA) was conducted, followed by Bonferroni test at a significance level of *P* < 0.05.

## 3 Results

### 3.1 The effects of Reducose®, 1-deoxynojirimycin, and l-leucine on glucose-induced insulin secretion in INS-1 cells

**Fig. 2:**
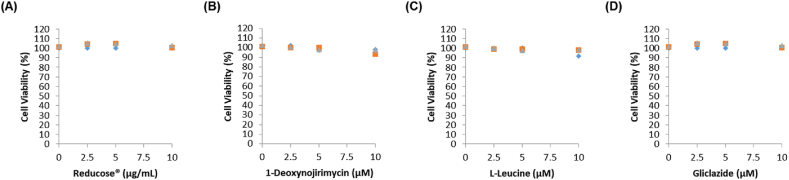
The effects of Reducose®, 1-deoxynojirimycin, and l-leucine on the viability of INS-1 cells. The effect of (A) Reducose®, (B) 1-deoxynojirimycin, (C) l-leucine, and (D) gliclazide on the viability of INS-1 cells following 24 h of incubation, compared to that of the control (0 μM), as determined by cell viability assays. n = 3 independent experiments, Kruskal–Wallis non-parametric test. The data are presented as the mean ± SEM.

**Fig. 3:**
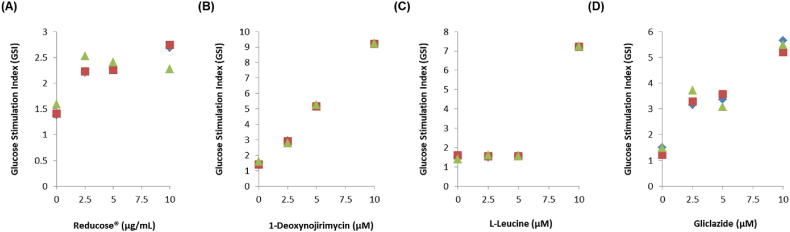
The effects of Reducose®, 1-deoxynojirimycin, and l-leucine on GSIS in INS-1 cells. The effect of (A) Reducose®, (B) 1-deoxynojirimycin, (C) l-leucine, and (D) gliclazide on the GSIS in INS-1 cells following 1 h of treatment, compared to that of the control (0 μM), as determined using the GSIS assay. The comparison of GSIS is expressed as fold-stimulation in terms of the GSI (16.7 mM glucose over 2.8 mM glucose for 1 h). n = 3 independent experiments, Kruskal–Wallis non-parametric test. The data are presented as the mean ± SEM.

A cell viability assay was performed to analyze the nontoxic concentrations of Reducose®, 1-deoxynojirimycin, and l-leucine in INS-1 cells. Treatment with Reducose® at concentrations of 2.5, 5, and 10 μg/mL, and treatment with 1-deoxynojirimycin, and l-leucine at concentrations of 2.5, 5, and 10 μM were observed to be nontoxic to INS-1 cells (Fig. 2A–D). Their non-toxic concentrations were tested to determine if they led to an increase in GSIS. As shown in Fig. 3A, the resultant GSI values were found to be 6.52 ± 0.13, 7.41 ± 0.31, and 12.26 ± 0.23 for Reducose® at 2.5, 5, and 10 μg/mL, respectively. As shown in Fig. 3B, the resultant GSI values were found to be 2.78 ± 0.31, 5.24 ± 0.21, and 9.23 ± 0.21 for 1-deoxynojirimycin at 2.5, 5, and 10 μM, respectively. As shown in Fig. 3C, the resultant GSI values were noted to be 7.56 ± 0.11 for l-leucine at 10 μM. As shown in Fig. 3D, the resultant GSI values were found to be 3.25 ± 0.13, 3.85 ± 0.21, and 5.65 ± 0.23 for gliclazide at 2.5, 5, and 10 μM, respectively. The results suggested that Reducose®, 1-deoxynojirimycin, and l-leucine enhanced insulin secretion in response to high glucose without exhibiting toxicity to INS-1 cells. Additionally, they increased the GSI values more effectively than gliclazide (positive control).

### 3.2 The effects of Reducose®, 1-deoxynojirimycin, and l-leucine on the protein expression of IRS-2 (Ser731), P-IRS-2, PPARγ, and PDX-1

**Fig. 4:**
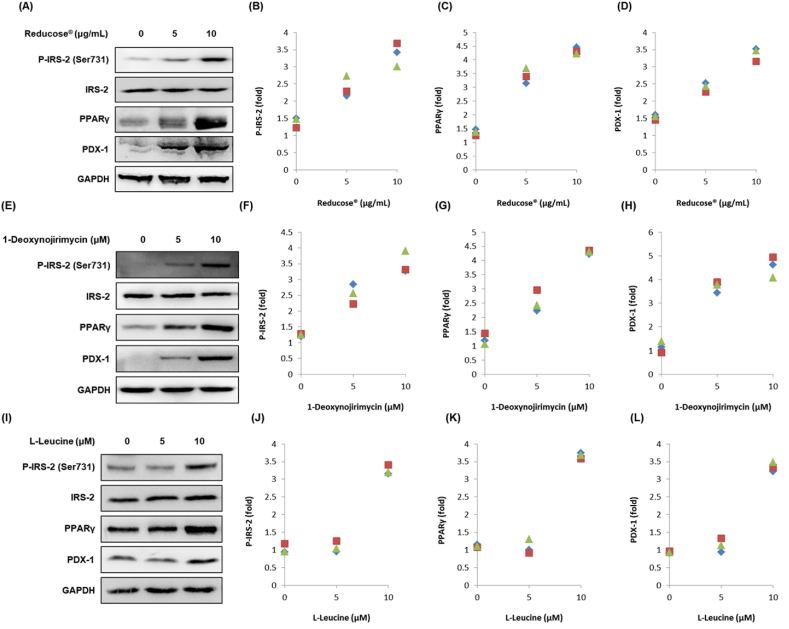
The effects of Reducose®, 1-deoxynojirimycin, and l-leucine on the protein expression levels of IRS-2 (Ser731), P-IRS-2, PPARγ, and PDX-1 in INS-1 cells. The protein expression levels of P-IRS-2 (Ser731), IRS-2, PDX-1, and GAPDH in INS-1 cells treated or untreated with (A–D) Reducose®, (E–H) 1-deoxynojirimycin, and (I–L) l-leucine for 24 h. Each bar graph presents the densitometric quantification of the Western blot bands. n = 3 independent experiments.

**Fig. 5:**
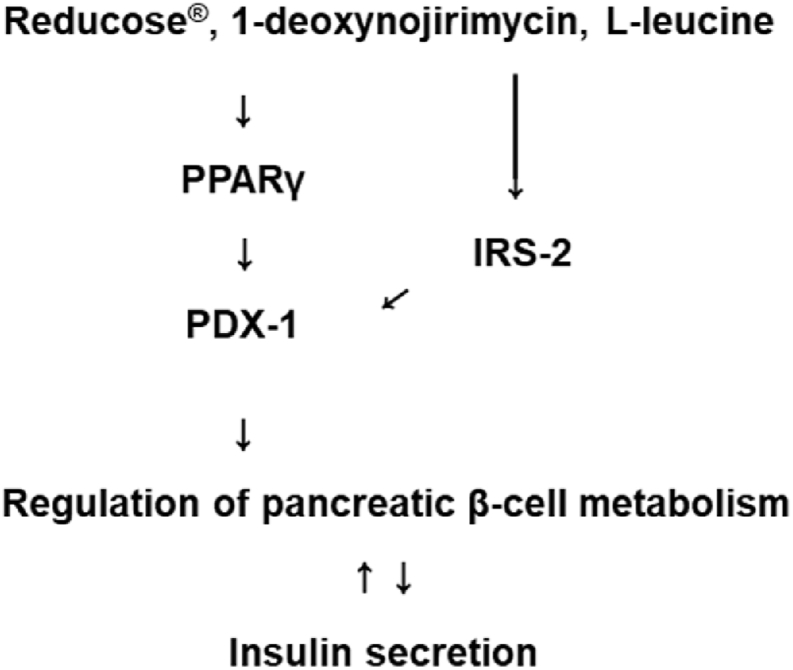
Schematic illustration of the effects of Reducose®, 1-deoxynojirimycin, and l-leucine on the protein expression levels of PPAR-γ, PDX-1, and IRS-2 in INS-1 cells.

Western blotting was performed to examine the expression of IRS-2 (Ser731), P-IRS-2, PPARγ, and PDX-1. Treatment with Reducose®, 1-deoxynojirimycin, and l-leucine increased the expression of these proteins compared to the untreated controls in INS-1 cells (Fig. 4A–L). This suggested that these compounds were effective in upregulating cellular signals related to insulin secretion (Fig. 5).

### 3.3 Changes in blood glucose levels after oral administration of Reducose®

The effect of oral administration of Reducose® on diabetic animal were assessed for 9 weeks as described in Fig. 1. Fasting blood glucose levels were checked at 3rd and 6th weeks to confirm the effect of Reducose® during the entire experimental period. In the 3rd week, the HFD/STZ group showed a significant increase in blood glucose levels (157.38 ± 37.52 mg/dL) compared to the NC group (89.60 ± 10.45 mg/dL), indicating elevated blood glucose levels. However, the MF/HFD/STZ group exhibited a significant decrease (110.88 ± 9.28 mg/dL) compared to the HFD/STZ group, indicating a notable reduction in blood glucose levels (Table 1). Furthermore, the R1/HFD/STZ, R3/HFD/STZ, and R6/HFD/STZ groups showed a significant reduction of over 20 % in blood glucose levels compared to the HFD/STZ group. In the 6th week, the concentration of fasting blood glucose showed a significant increase in the HFD/STZ group (176.38 ± 12.84 mg/dL) compared to the NC group (106.20 ± 24.19 mg/dL). The MF/HFD/STZ group also exhibited an increase (133.38 ± 14.52 mg/dL) compared to the third-week blood glucose levels. Additionally, the R1/HFD/STZ, R3/HFD/STZ, and R6/HFD/STZ groups demonstrated a dose-dependent significant decrease in blood glucose levels compared to the HFD/STZ group. Among them, the R6/HFD/STZ group showed lower blood glucose levels than the positive control group (Table 1). According to these results, it was confirmed that Reducose® exhibited an inhibitory effect on blood glucose increase rapidly from 3 weeks after oral administration.

**Table 1:** Effect of Reducose® on blood glucose levels of HFD/STZ-induced diabetic rats.

Group	Glucose level (mg/dL)		
	Initial	3 week	6 week
NC	94.00 ± 12.51	89.60 ± 10.45	106.20 ± 24.19
HFD/STZ	157.88 ± 33.52^##^	157.38 ± 37.52^##^	176.38 ± 12.84^##^
MF/HFD/STZ	157.25 ± 28.73^##^	110.88 ± 9.28**	133.38 ± 14.52**
R1/HFD/STZ	155.75 ± 27.34^##^	119.63 ± 22.96*	137.75 ± 16.31**
R3/HFD/STZ	157.13 ± 23.40^##^	119.69 ± 18.37*	135.63 ± 25.34**
R6/HFD/STZ	156.38 ± 22.39^##^	119.63 ± 23.98*	129.44 ± 12.17**

NC, normal control; HFD/STZ, high fat diet + diabetic control; MF/HFD/STZ, metformin 200 mg/kg + high fat diet + diabetic; R1/HFD/STZ, Reducose® 15 mg/kg + high fat diet + diabetic; R3/HFD/STZ, Reducose® 30 mg/kg + high fat diet + diabetic; R6/HFD/STZ, Reducose® 60 mg/kg + high fat diet + diabetic. Values are expressed as means ± SEM. ^##^*P* < 0.01 (compared with NC) and *P < 0.05, **P < 0.01 (compared with HFD/STZ).

### 3.4 Effect of Reducose® on oral glucose tolerance test

**Fig. 6:**
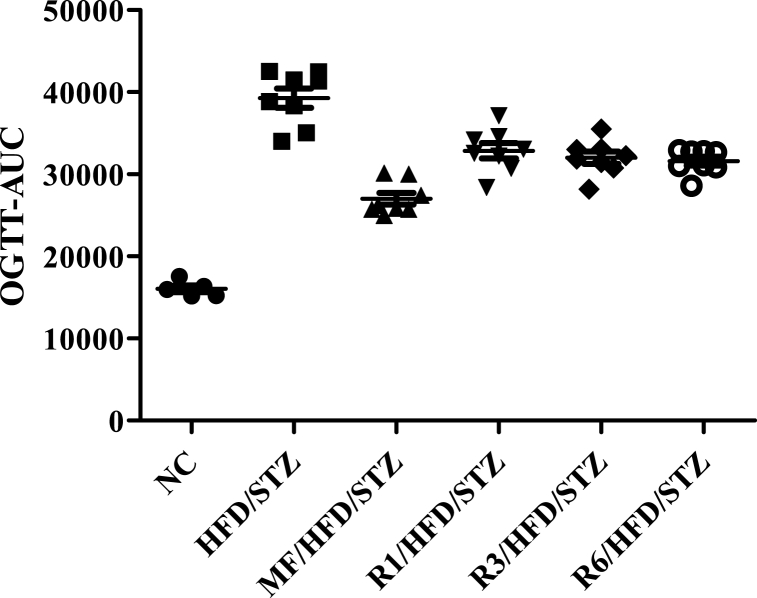
Area under the curve of OGTT in each group. NC, normal control; HFD/STZ, high fat diet + diabetic control; MF/HFD/STZ, metformin 200 mg/kg + high fat diet + diabetic; R1/HFD/STZ, Reducose® 15 mg/kg + high fat diet + diabetic; R3/HFD/STZ, Reducose® 30 mg/kg + high fat diet + diabetic; R6/HFD/STZ, Reducose® 60 mg/kg + high fat diet + diabetic. n = 8 independent experiments. Values are expressed as means ± SEM.

The effects of administering Reducose® on oral glucose tolerance in diabetic rats were observed by orally administering a high concentration of glucose. The blood glucose levels and oral glucose tolerance test-area under the curve (OGTT-AUC) results at specific time points (0, 30, 60 and 120 min) were recorded and presented in Fig. 6. Compared to the NC group (16059.00 ± 964.83), the HFD/STZ group showed a significant increase in OGTT-AUC with a value of 39264.38 ± 3304.42. In contrast, the MF/HFD/STZ (27009.38 ± 2028.69), R1/HFD/STZ (32865.00 ± 2626.18), R3/HFD/STZ (32019.38 ± 2110.59), and R6/HFD/STZ (31599.38 ± 1507.46) groups exhibited a significant decrease in OGTT-AUC compared to the HFD/STZ group.

### 3.5 Effect of Reducose® on AST, ALT, TG and TC levels

The analysis of liver parameters and serum lipid levels in the positive control group and the groups administered with Reducose® is presented in Table 2. In terms of AST, it was observed that the values increased in the diabetic groups compared to the NC group, but there were no significant differences among the treatment groups compared to the HFD/STZ group. However, a decrease was observed in the treatment groups. Similarly, for ALT and TG, an increase was observed in the diabetic groups compared to the NC group, but the values were lower compared to the HFD/STZ group. Regarding TC, the HFD/STZ group showed a significant increase compared to the NC group, whereas a decrease was observed with the oral administration of metformin and Reducose®.

**Table 2:** Effect of Reducose® on serum levels in HFD/STZ-induced diabetic rats.

Group	AST	ALT	TG	TC
	U/L	U/L	mg/dL	mg/dL
NC	57.80 ± 6.76	19.75 ± 2.63	51.80 ± 20.36	57.80 ± 8.61
HFD/STZ	85.88 ± 46.42	36.88 ± 18.04	79.29 ± 8.54^##^	84.63 ± 12.26^##^
MF/HFD/STZ	62.43 ± 6.32	29.57 ± 7.44	73.25 ± 12.21^#^	74.25 ± 14.23
R1/HFD/STZ	69.88 ± 15.52	31.63 ± 8.99	72.38 ± 4.98^#^	84.38 ± 5.04^##^
R3/HFD/STZ	67.88 ± 15.36	32.50 ± 14.01	73.75 ± 7.05^#^	77.50 ± 12.49
R6/HFD/STZ	58.14 ± 5.52	31.88 ± 9.22	72.38 ± 12.34^#^	76.38 ± 16.31

NC, normal control; HFD/STZ, high fat diet + diabetic control; MF/HFD/STZ, metformin 200 mg/kg + high fat diet + diabetic; R1/HFD/STZ, Reducose® 15 mg/kg + high fat diet + diabetic; R3/HFD/STZ, Reducose® 30 mg/kg + high fat diet + diabetic; R6/HFD/STZ, Reducose® 60 mg/kg + high fat diet + diabetic. AST, aspartate transaminase; ALT, alanine transaminase; TG, triglyceride; TC, total cholesterol. Values are expressed as means ± SEM. ^#^*P* < 0.05, ^##^*P* < 0.01 (compared with NC).

### 3.6 Effect of Reducose® on glucose and insulin levels

The impact of Reducose® administration on serum glucose and insulin levels was measured, and the results are summarized in Table 3. In terms of glucose, the HFD/STZ group showed a significant increase at 241.75 ± 74.00 mg/dL compared to the NC group at 153.20 ± 7.01 mg/dL. However, the MF/HFD/STZ group at 165.75 ± 11.34 mg/dL and the R6/HFD/STZ group at 150.71 ± 5.94 mg/dL demonstrated significant reductions in blood glucose levels compared to the HFD/STZ group. As for insulin, the NC group exhibited higher insulin levels at 5.22 ± 0.66 ng/mL compared to the HFD/STZ group at 2.03 ± 0.66 ng/mL. The administration of metformin and Reducose® resulted in significant increases in insulin levels compared to the HFD/STZ group.

**Table 3:** Effect of Reducose® on glucose and insulin levels in HFD/STZ-induced diabetic rats.

Group	Glucose	Insulin
	mg/dL	ng/mL
NC	153.20 ± 7.01	5.22 ± 0.66
HFD/STZ	241.75 ± 74.00^##^	2.03 ± 0.66^###^
MF/HFD/STZ	165.75 ± 11.34**	4.67 ± 0.59***
R1/HFD/STZ	190.75 ± 39.89	3.41 ± 0.85^##,^*
R3/HFD/STZ	158.14 ± 11.77**	4.04 ± 1.09***
R6/HFD/STZ	150.71 ± 5.94***	3.74 ± 0.61^#,^**

NC, normal control; HFD/STZ, high fat diet + diabetic control; MF/HFD/STZ, metformin 200 mg/kg + high fat diet + diabetic; R1/HFD/STZ, Reducose® 15 mg/kg + high fat diet + diabetic; R3/HFD/STZ, Reducose® 30 mg/kg + high fat diet + diabetic; R6/HFD/STZ, Reducose® 60 mg/kg + high fat diet + diabetic. Values are expressed as means ± SEM. ^#^*P* < 0.05, ^##^*P* < 0.01, ^###^*P* < 0.001 (compared with NC), *P < 0.05, **P < 0.01, ***P < 0.001 (compared with HFD/STZ).

## 4 Discussion

In the present study, the insulin releasing activities of Reducose®, 1-deoxynojirimycin, and l-leucine were evaluated by measuring the GSIS, which was expressed as GSI. Reducose®, 1-deoxynojirimycin, and l-leucine enhanced insulin secretion in response to high glucose without exhibiting toxicity to INS-1 cells, and also increased the GSI values more effectively than gliclazide (positive control). Several studies have reported that mulberry leaf extract (Moraceae*, Morus alba* L.) displays anti-diabetic activity. In diabetic rats, polysaccharides and fagomine from mulberry leaves increased insulin secretion and protein expression of PDX-1 in pancreatic β-cells [19,20]. Additionally, 1-deoxynojirimycin, an active ingredient in mulberry leaf extract, enhances the potential of insulin secretion [21,22]. Additionally, it improved insulin sensitivity in diabetic rats and mice [23,24]. In humans, mulberry powder, which contains the highest 1-deoxynojirimycin content, reduced the rise in postprandial blood sugar [25]. In addition, l-leucine has been linked to increased insulin secretion both in vivo and in vitro [26,27]. However, detailed mechanistic studies on insulin secretion by pancreatic-cells are lacking.

To evaluate the potential effect of Reducose®, 1-deoxynojirimycin, and l-leucine on insulin secretion, we performed Western blot assays to detect protein expression related to pancreatic β-cell functions. In diabetic rats, polysaccharides and 1-deoxynojirimycin from mulberry leaves increase insulin secretion and protein expression of PDX-1 in pancreatic-cells [19,24]. Consistent with previous studies, in the present study, treatment with Reducose®, 1-deoxynojirimycin, and l-leucine enhanced the expression of PDX-1. The islet transcription factor PDX-1 is important for pancreatic β-cells [28]. It has previously been demonstrated that PDX-1 deficiency in mouse pancreatic β-cells impairs GSIS [29]. PDX-1 is a transcriptionally regulated target gene for PPAR-γ in pancreatic β-cells [30]. Previous studies indicate that PPAR-γ antagonists inhibit GSIS, whereas PPAR-γ agonists enhance GSIS in INS-1 cells [31]. In the present study, the treatment of Reducose®, 1-deoxynojirimycin, and l-leucine enhanced the expression of PPAR-γ. IRS-2 signaling, an upstream activator of PDX-1, has been implicated in the synthesis and secretion of insulin. This role has been reported in the in vivo study that showed that GSIS is reduced in β cell-specific IRS-2-knockout mice [32]. Here, we provide data to suggest that IRS-2 was the upstream activator of PDX-1 that responded to the treatment of Reducose®, 1-deoxynojirimycin, and l-leucine. In conclusion, we demonstrated that treatment with Reducose®, 1-deoxynojirimycin, and l-leucine enhanced GSIS by regulating PDX-1 expression via PPAR-γ and IRS-2 signaling in INS-1 cells. This may play an important role in mediating normal pancreatic cell metabolism and in increasing GSIS. These results suggested that Reducose® play a role in promoting GSIS but not enough to show the detailed mechanistic studies. However, increases in GSIS by regulating PDX-1 expression via PPAR-γ and IRS-2 is valuable as fundamental data for detailed mechanistic studies. Impaired GSIS is responsible for pancreatic β-cell failure. PDX-1 expression via PPAR-γ and IRS-2 signaling plays a central role in pancreatic β-cell function and survival [33]. These results were similar to the mechanism of action in our previous experimental results of methyl caffeate [34].

As a result of animal experiments, significant effects were shown from the middle dose (30 mg/kg). A medium dose of 30 mg/kg is about 250 mg when converted to a clinical dose in consideration of the body mass index, and this concentration is also frequently used in clinical trials. However, it is difficult to predict accurate clinical efficacy only with cell and animal test results, and additional various reviews are needed. Additional consideration is needed for pharmacokinetic studies of active ingredients and side effects that have not yet been reviewed.

## 5 Conclusion

In summary, GSIS assays led to the discovery of Reducose®, 1-deoxynojirimycin, and l-leucine, which significantly increased GSIS of β-cells without cytotoxicity. Western blot assays revealed that Reducose®, 1-deoxynojirimycin, and l-leucine could augment GSIS by regulating the expression of proteins, including P-IRS-2, PDX-1, and PPARγ. Additionally, Reducose® treatment can not only decrease AST, ALT, TG, and TC levels in T2DM rats but also reduce glucose and insulin levels. Therefore, Reducose®, 1-deoxynojirimycin, and l-leucine may be useful as candidates for the treatment of T2DM.

## Ethics statement

The study was approved by institutional animal care and use committees of Gachon University (GU1-2022-IA0068).

## Funding statement

This research was supported by the National Research Foundation of Korea (NRF) funded by the Ministry of Science & ICT (NRF-2021M3H9A1097888).

## Additional information

No additional information is available for this paper.

## Data availability statement

All data underlying the results are available as part of the article and no additional source data are required.

## CRediT authorship contribution statement

**Dahae Lee:** Investigation. **Ji Yun Baek:** Formal analysis. **Ye Jung Choi:** Formal analysis. **Min Ji Han:** Formal analysis. **Seon Hwa Kim:** Formal analysis. **Tae Hoon Kim:** Formal analysis. **Sanghyun Lee:** Investigation. **Ki Sung Kang:** Project administration.

## Declaration of competing interest

The authors declare that they have no known competing financial interests or personal relationships that could have appeared to influence the work reported in this paper.
