# Supplementary material for: Glucose-lowering effect of Reducose® enriched with 1-deoxynojirimycin and l-leucine: Studies on insulin secretion in INS-1 cells and reduction of blood glucose in diabetic rats
Source: Heliyon. 2024 Feb 1;10(3):e25499. doi: 10.1016/j.heliyon.2024.e25499 (PMC10850582; doi:10.1016/j.heliyon.2024.e25499)
Supplement: Multimedia component 1 [file mmc1.docx]

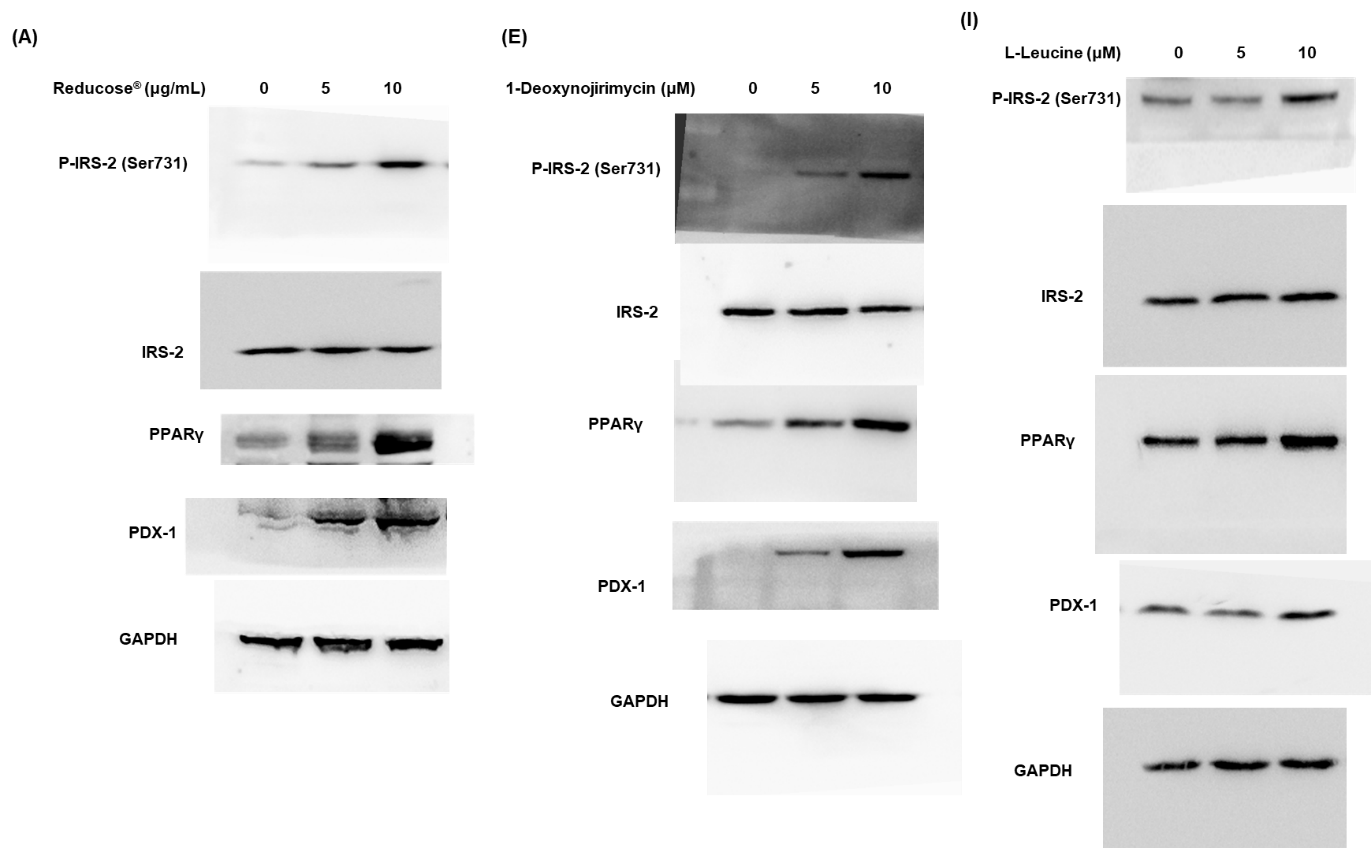


Figure 3. The effects of reducose, 1-deoxynojirimycin, and L-leucine on the protein expression levels of insulin receptor substrate-2 (IRS-2) (Ser731), P-IRS-2, peroxisome proliferator-activated receptor-γ (PPARγ), and pancreatic and duodenal homeobox-1 (PDX-1) in INS-1 cells. Protein expression levels of P-IRS-2 (Ser731), IRS-2, PDX-1, and glyceraldehyde 3-phosphate dehydrogenase (GAPDH) in INS-1 cells treated or untreated with (A–D) reducose, (E–H) 1-deoxynojirimycin, and (I–L) L-leucine for 24 h. Each bar graph presents the densitometric quantification of western blot bands. **p* < 0.05 compared to the control (0 μM).
